# Case report: Long-term survival in puppies assessed with echocardiography, electrocardiography and cardiac troponin I after acute death in littermates due to parvoviral myocarditis

**DOI:** 10.3389/fvets.2023.1229756

**Published:** 2023-08-14

**Authors:** Brenda Dines, Heidi Kellihan, Carolyn Allen, Alan Loynachan, Philip Bochsler, Sandra Newbury

**Affiliations:** ^1^Department of Medical Sciences, University of Wisconsin-Madison School of Veterinary Medicine Shelter Medicine Program, Madison, WI, United States; ^2^Department of Medical Sciences, University of Wisconsin-Madison School of Veterinary Medicine, Madison, WI, United States; ^3^Department of Veterinary Science, University of Kentucky Veterinary Diagnostic Laboratory, Lexington, KY, United States; ^4^Department of Pathobiological Sciences, Wisconsin Veterinary Diagnostic Laboratory, University of Wisconsin-Madison School of Veterinary Medicine, Madison, WI, United States

**Keywords:** parvo, myocarditis, puppy, troponin (cTnI), survival, prognosis, parvovirus, CPV-2

## Abstract

Positive clinical outcomes of a group of surviving puppies from a litter affected by parvoviral myocarditis are detailed in this case report. Past reports focus on the negative outcomes of littermates of puppies who have died of parvoviral myocarditis. In this case, two puppies in a shelter setting, from a litter exposed to parvovirus, died suddenly with parvoviral myocarditis diagnosed at necropsy. The other seven puppies were screened for cardiac health with echocardiogram, electrocardiogram, and cardiac troponin I prior to adoption. All seven puppies had normal echocardiograms, electrocardiograms, and normal initial and recheck cardiac troponin I results. At recheck 2 years after the initial round of testing, two of the puppies were screened and continue to have normal cardiac diagnostics. All seven dogs are alive and thriving at 5 years old in homes with adopters who were given a complete medical history on the dogs prior to adoption. In summary, the outcomes for puppies in litters affected by parvoviral myocarditis are variable but they do not have to be grave. The use of cardiac diagnostics including echocardiogram, electrocardiogram and cardiac troponin I may serve as a prognostic basis for assessing the potential outcomes for the surviving puppies in affected litters.

## 1. Introduction

The present case report describes a litter of puppies who were admitted to an animal shelter who had a history of perinatal exposure to parvovirus. Two of the littermates died suddenly secondary to parvoviral myocarditis (PMC). The seven surviving littermates were evaluated for evidence of cardiac dysfunction through echocardiography, electrocardiography (ECG), and cardiac troponin I (cTnI) prior to adoption. The puppies evaluated have since survived almost 5 years.

Canine parvovirus 2 (CPV-2) is a non-enveloped DNA virus that has been reported to cause both severe enteritis and myocarditis in dogs (1–5). Puppies who are infected by CPV-2 in the perinatal period may develop PMC (6, 7). Parvoviruses typically infect rapidly dividing cells. In puppies, the myocardial cells are rapidly dividing in the 2 weeks just after birth and it is during this time that infection with CPV-2 can result in myocarditis (8). While reports of myocarditis are rare, parvoviral enteritis remains a significant cause of morbidity and mortality in dogs despite development of an effective vaccine (9, 10).

Previous literature suggests a grave prognosis for puppies within litters affected by PMC. Entire litters or large portions of litters affected by myocarditis were reported to have poor outcomes including sudden death in the weeks after birth or a decline into heart failure in the months after birth. Information about longer term positive outcomes for puppies in affected litters is very limited, leaving a gap in information concerning long-term outcomes (3, 5, 9, 11–15).

The objectives of reporting this case are to demonstrate (1) the use of echocardiography, ECG, and cTnI as potential prognostic indicators for surviving littermates of puppies who have died of PMC, (2) that survival is variable and prognosis may be good for littermates of puppies with PMC, and (3) that despite being fully informed of the potential for poor prognosis, people were still willing to adopt these puppies.

## 2. Patient information

**Figure 1:**
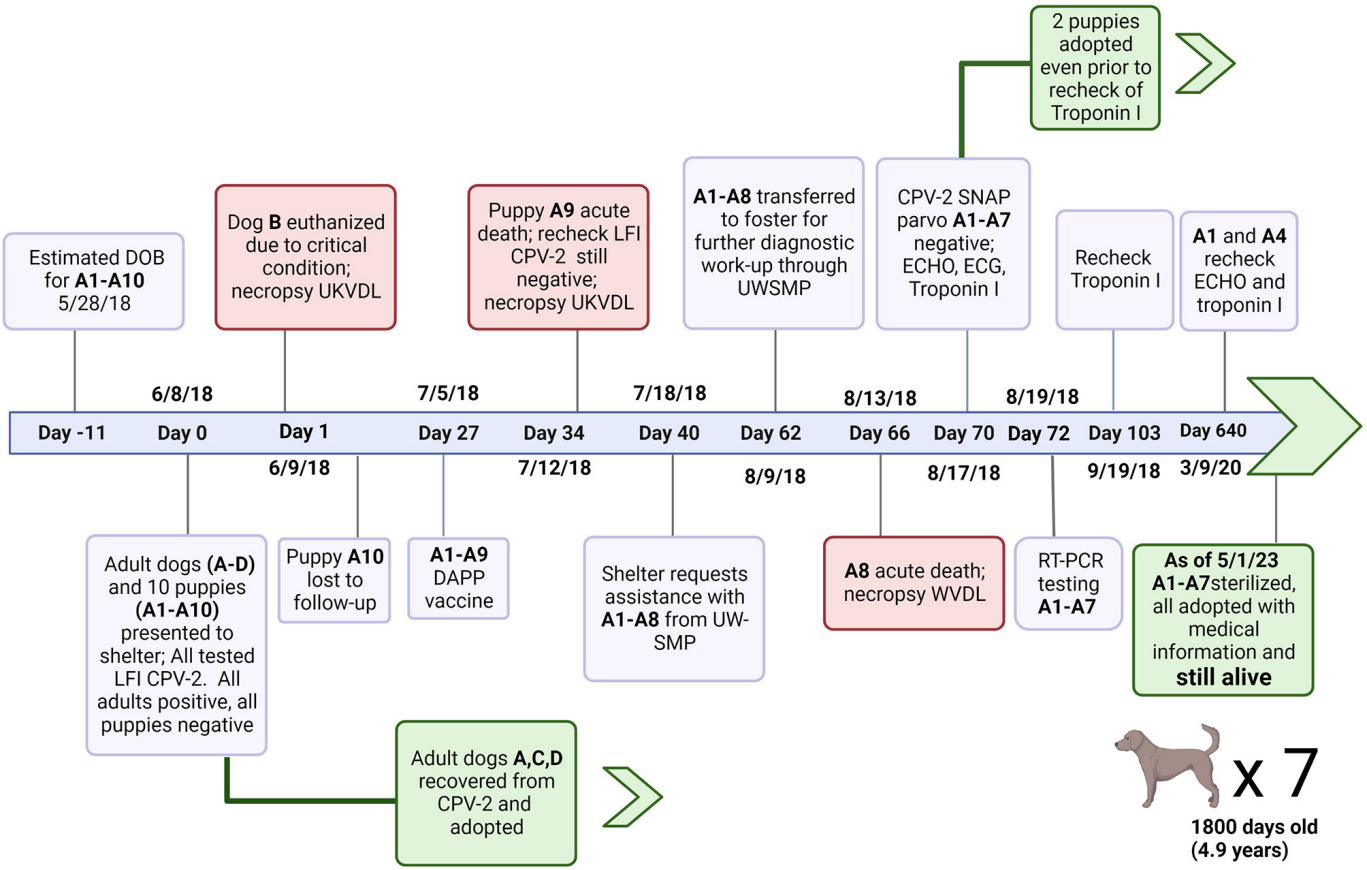
Timeline of events from initial perinatal exposure to CVP-2 to shelter outcomes for puppies A1–A10. Canine Parvovirus type 2 (CPV-2); Lateral flow immunochromatographic assay (LFI); University of Kentucky Veterinary Diagnostic Laboratory; canine distemper virus, canine adenovirus type 1 and 2, canine parainfluenza virus, and canine parvovirus type 1 and 2 vaccine (DAPP); University of Wisconsin Shelter Medicine Program (UWSMP); RT-PCR (Real time polymerase chain reaction); SNAP™ parvo (SNAP parvovirus antigen test kit, IDEXX laboratories); echocardiogram (ECHO); electrocardiogram (ECG). Created with Biorender.com.

Eight large mixed breed puppies (A1–A8) from a litter of 10 were transported to a rescue group in early June 2018 after one pup from their litter died acutely (A9). The litter was initially part of a larger group of dogs admitted to an animal shelter including two intact males (Dog C and D) and two intact females (Dog A and B). Dog A was actively nursing the litter of ten puppies, six females (A1–A5) and four males (A6–A10), estimated to be one to two weeks old (A1–A10) (Figure 1).

No clinical signs of illness were reported for any of the 10 puppies (A1–A10) who were examined by lay staff at shelter admission (Day 0). The four adults (A, B, C, and D) were also examined on Day 0. Clinical signs reported were similar in all adults and included vomiting, diarrhea, and lethargy consistent with CPV-2 infection. Rectal swab samples from all dogs and puppies in the group were tested individually at intake with a lateral flow immunochromatographic (LFI) test for CPV-2 antigen. Dogs A, B, C and D tested positive for CPV-2 antigen, while puppies A1–A10 tested negative.

Dog B was euthanized the day of intake due to the severity of clinical signs and submitted for necropsy which confirmed severe parvoviral enteritis. The remaining three adult dogs and puppies were kept separated from the general population. Adults were treated with supportive care, and recovered uneventfully (Figure 1). None of the puppies developed clinical signs of parvoviral enteritis, and so required no treatment.

One puppy, A10, was taken from the shelter without record and was lost to follow up. Puppies were monitored daily and were reportedly thriving in the shelter. Routine preventative care was provided including the parental administration of a modified live Distemper-Adenovirus-Parvovirus-Parainfluenza vaccine on Day 27.

No clinical signs of illness were reported in the litter until A9 was found deceased 34 days after intake. A repeat LFI parvovirus antigen test returned a negative result from the rectal swab of A9.

**Figure 2:**
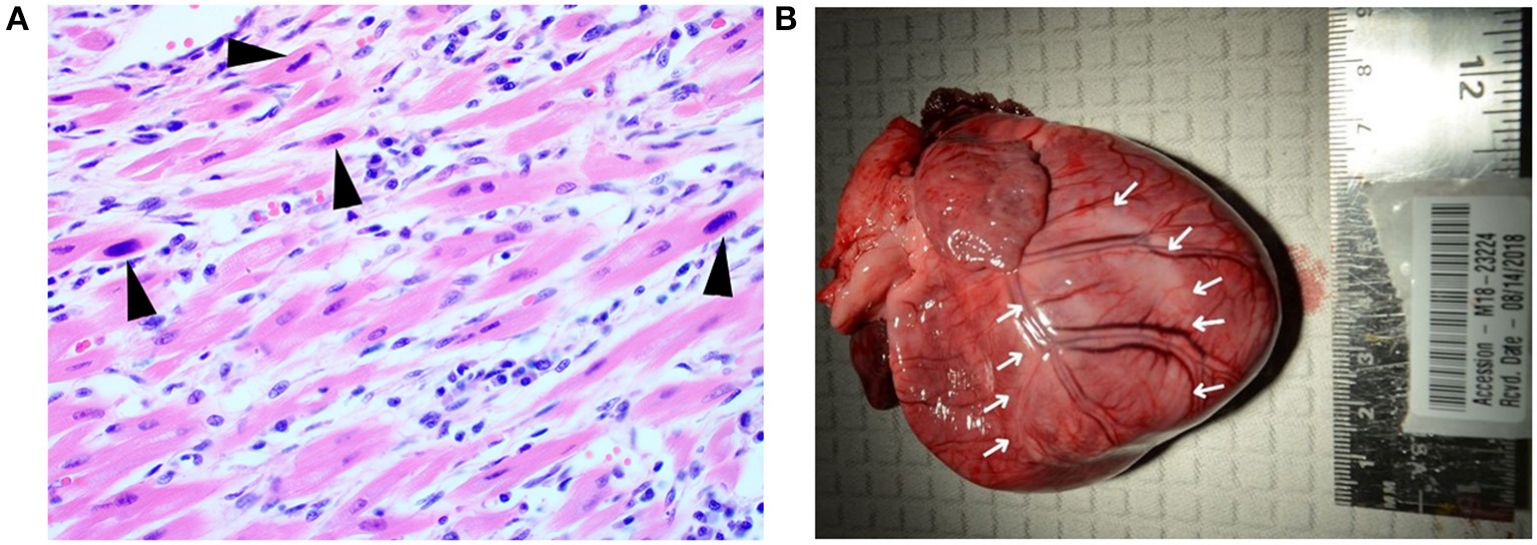
Histopathological and gross cardiac findings associated with acute parvoviral myocarditis and post-myocarditis DCM phenotype. **(A)** Puppy A9. Intranuclear viral inclusion bodies (black arrowheads) are located in cardiomyocytes. The adjacent interstitium is expanded by low numbers of fibroblasts, lymphocytes, and plasma cells. Hematoxylin and eosin stain. 400× magnification. **(B)** Puppy, A8, heart. The left ventricle is pale (denoted by white arrows). Pallor extends over the region of the interventricular septum to the right ventricle.

Bilateral pulmonary congestion was noted on postmortem examination of A9 at University of Kentucky Veterinary Diagnostic Laboratory. Upon histopathologic examination, multifocal to coalescing regions of the myocardium were expanded by minimal to small streams of collagenous connective tissue that was infiltrated with low to moderate numbers of lymphocytes and plasma cells. Large, basophilic, intranuclear viral inclusion bodies were multifocally located in cardiomyocytes (Figure 2). Findings in the lungs and intestines were unremarkable. The pathologic diagnosis for A9 was severe chronic multifocal to coalescing lymphoplasmacytic myocarditis with intranuclear viral inclusion bodies. Necropsy findings were consistent with death due to PMC. A CPV-2 fluorescent antibody test was performed on tissues, but was negative. However, this was considered a false negative result because heart was not collected or tested by the fluorescent antibody test procedure.

Because previous literature suggested a potentially grave prognosis for the remaining littermates a transfer was arranged to facilitate foster care and assessment by the author. Cardiac work-up was planned if the puppies were deemed healthy on initial physical exam.

### 2.1. Clinical findings

Post-transport, no abnormalities were found on general physical and cardiovascular examinations of A1–A8. Four days after initial presentation (Day 66), puppy A8 was found dead after being observed and apparently healthy 2 h earlier.

### 2.2. Diagnostic assessment

Diagnostic assessment included pathologic evaluation of animals who died. Diagnostic testing for living animals included PCR and ELISA testing for CPV-2, echocardiography, ECG, and cTnI.

#### 2.2.1. Pathology

Postmortem examination at Wisconsin Veterinary Diagnostic Laboratory for Puppy A8 revealed cardiac dilatation with multifocal and locally extensive areas of myocardial pallor in the left and right ventricles (Figure 2). Cut surfaces of the left and right ventricular free walls also exhibited pallor. The left ventricular free wall was thin, measuring 5 mm in width. The right ventricular free wall was also thin, measuring 2.5 mm in width. The heart was 1.33% of the body weight while ~0.7%−0.8% of the body weight is considered normal in adult and neonatal dogs (16). There was ~5 ml of serosanguinous fluid in both the thoracic and abdominal cavities.

Significant histologic findings commonly seen with heart failure were present in the lungs, liver and heart. The lungs displayed moderate alveolar histiocytosis. The liver had moderate centrilobular congestion and moderate lipid-type hepatocellular vacuolar change. The heart exhibited severe, chronic, multifocal and locally extensive myofiber atrophy, loss, and replacement with fibrous connective tissue. Low to moderate numbers of lymphoplasmacytic inflammatory cells infiltrated the myocardium.

A section of the cardiac ventricular wall which exhibited pallor was strongly positive for CPV-2 on real time PCR with a 24.3 cycle threshold (Ct). Although no histologic changes were present in the intestines, samples from that tissue were strongly positive on real time PCR (Ct = 24.0) as well.

Results of the necropsy findings supported PMC as a cause of death for A8.

#### 2.2.2. Parvo RT-PCR

Samples were collected and tested by RT-PCR for each of the remaining puppies on Day 62 (Table 1).

**Table 1:** Diagnostic results for A1–A7.

**Patient**	**CPV-2 RT-PCR (Ct) (Day 72)**	**CPV-2 ELISA (Day 70)**	**cTnI (ng/ml) (Day 70)**	**cTnI (ng/ml) (Day 103)**	**cTnI (ng/ml) (Day 640)**
A1	Negative	Negative	0.06	0.02	0.02
A2	39	Negative	0.06	0.03	NT
A3	Negative	Negative	0.02	0.02	NT
A4	32.8	Negative	0.02	< 0.01	< 0.01
A5	38.2	Negative	0.06	0.03	NT
A6	34.6	Negative	0.02	0.02	NT
A7	37.3	Negative	0.01	< 0.01	NT

CPV-2 RT-PCR, Canine Parvovirus 2 Real Time PCR; Ct, cycle threshold; CPV-2 ELISA, canine parvovirus 2 ELISA; cTnI, cardiac troponin I; NT, not tested.

Reference range for dogs < 0.006–0.17 ng/ml (cardiac troponins in dogs and[[Inline Image]] cats, Langhorn and Willesin (17)).

#### 2.2.3. CPV-2 rapid ELISA antigen

One week later, on Day 70, on initial presentation at University of Wisconsin Veterinary Teaching Hospital, rectal swabs were collected from each puppy (A1–A7) for in house testing and submitted for a CPV-2 rapid ELISA antigen. Results are presented in Table 1.

#### 2.2.4. Echocardiography

The puppies (11 weeks old) were then presented to the University of Wisconsin Veterinary Cardiology service. Each puppy was given a brief exam and sedated with 0.5 mg/kg butorphanol intramuscularly to facilitate handling during cardiac diagnostics. Each puppy was auscultated right before injection. Echocardiography (Vivid E95 Echocardiographic System, General Electric, Waukesha, WI, USA) was performed in a standard fashion. Echocardiographic measurements (M-mode, 2-D, and Doppler) were all within normal limits for puppies A1–A7, with results provided in Supplementary Table S1.

**Figure 3:**
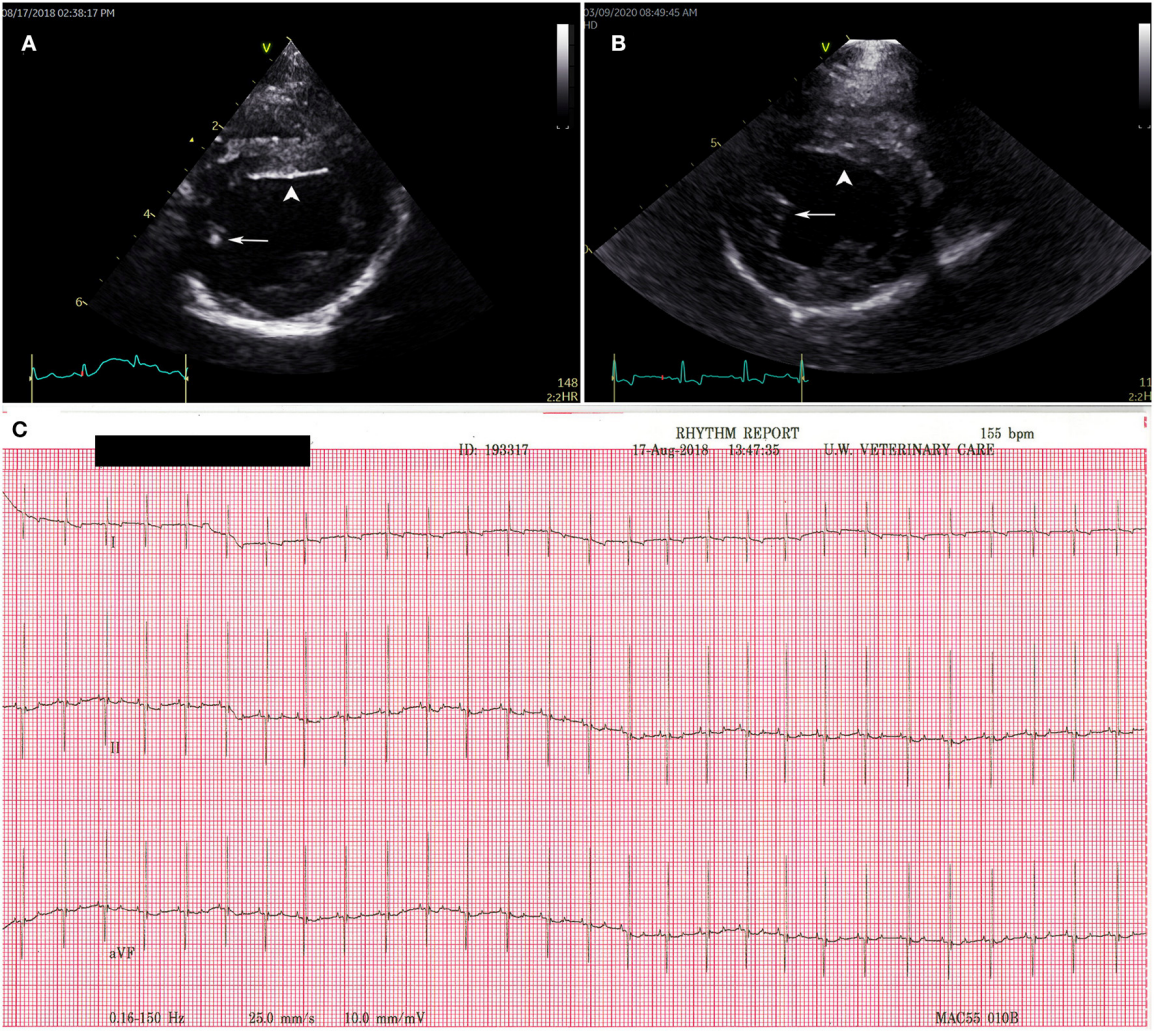
Transthoracic echocardiogram and electrocardiogram findings for puppy A4. Right parasternal short axis view at the level of the left ventricular papillary muscles **(A)** at 11 weeks old with a hyperechoic region in the posterior papillary muscle (white arrow) and on the endocardial surface of the interventricular septum (white arrow head). **(B)** Repeat of puppy A4 echocardiogram at 1.5 years of age. The hyperechoic regions (white arrow and white arrow head) have resolved. **(C)** Normal electrocardiography results for puppy A4 at 11 weeks. Puppies A1–3 and puppies 5–7 all had normal electrocardiograms consistent with the tracing presented for puppy A4.

Puppy A4 and puppy A6 both had hyperechoic areas in the left ventricle that could represent local ischemia at the time of their 11 week echocardiogram (Figure 3). Echocardiograms were otherwise normal for puppies A1–A7. Puppies A1 and A4 had a repeat echocardiograms performed just under 2 years later and the hyperechoic areas seen in the left ventricle of A4 at 11 weeks of age were no longer visible (Figure 3). There were no structural abnormalities noted on repeat echocardiograms for A1 and A4.

#### 2.2.5. Electrocardiography

Standard practices for ECG (MAC 5000 Electrocardiogram, General Electric, Waukesha, WI, USA) were followed for patients A1–A7. A normal sinus rhythm was reported in A1–A7, no arrythmia, conduction abnormalities, nor were ST segment changes noted (Figure 3).

#### 2.2.6. Cardiac troponin I

Upon completion of cardiac evaluation on day 70, blood was taken from the jugular vein of each pup and submitted for cTnI testing to University of Wisconsin Health. The veterinary cardiologist was blinded to the cTnI results until the echocardiograms were evaluated and a formal report submitted.

On day 103, a second sample was collected from the cephalic vein of each puppy and sent to University of Wisconsin Health for a recheck cTnI. Puppies A1 and A4 had a third sample taken for cTnI testing on day 640. As before, these samples were submitted to the University of Wisconsin Health. Results are listed in Table 1.

## 3. Discussion

This case report presents 5 years of positive follow-up for seven surviving puppies in a litter affected by PMC which is three times longer than has been previously reported (3). Previous publications regarding PMC have focused primarily on characterizing the disease in puppies who have not survived (2, 6, 11, 13, 18). The clinical information provided in this case report fills what has been a gap in information regarding the outcomes of the surviving littermates from litters affected by PMC (2, 3). The results of advanced cardiac diagnostics including echocardiogram, ECG, and cTnI are presented as a potential model for prognostication. We report willingness of the public to adopt puppies with transparency about potentially grave outcomes in hopes that it may encourage further investment in puppies, as appropriate to resources available, even when a litter has had puppies affected by PMC.

The variable outcomes of puppies in the litter described in this case report exemplify the importance of diagnostic necropsy in cases where sudden death affects a litter, particularly when exposure to parvovirus is suspected. Point of care testing was used three different times during this case report, and none of the fecal swab-based point of care tests (LFI and SNAP™ Parvo) could detect parvovirus infection in any of the puppies A1–A10. Even in A9 and A8, where each puppy had clear evidence of PMC on necropsy exam, as demonstrated by histological evidence of parvovirus infection in the heart (A9) or positive PCR results (A8), point of care fecal swab did not detect CPV-2. Previous reports demonstrate that infectious virus can be isolated from the hearts of puppies with PMC and that some puppies will only have infection of the myocardium rather than infection in both the heart and small intestine (6, 7). Since it is possible for puppies with PMC to not have parvovirus infection in the intestines, the use of point of care CPV-2 testing, in the absence of gastrointestinal clinical signs, may give a negative result but cannot exclude PMC. Infectious risk was considered very low since puppies had repeated negative point of care tests and no clinical signs (10).

When fecal PCR testing was performed, positive tests with high Ct (low viral load) values were reported for puppies A2 and A4–A7. The presence of a positive PCR test in this group of puppies may be related either to recent vaccination or infection with live virus but cannot be differentiated in this case (19, 20).

Puppies affected by PMC described in previous reports fit roughly into two categories: (1) puppies who die acutely in the first weeks of life (~2–7 weeks), and (2) puppies and dogs who die months after birth up until the second year of life (5, 11, 13, 15, 18, 21).

In this case report, consistent with what has been described for this first category, one pup died at 45 days old. Histopathology showed infection of cardiomyocytes with viral inclusion bodies with low numbers of lymphocytes and plasma cells signifying infection of cardiomyocytes with parvovirus and the inflammatory response to viral infection (Figure 2). Another pup died suddenly at day 77, consistent with what has been described for the second category (4, 11, 13, 14, 18). Necropsy findings for this puppy were consistent with the second category with chronic cardiac changes leading to congestive heart failure. These have been described previously as dilated cardiomyopathy phenotype (DCM phenotype) (22).

This report also identifies and follows a third category of puppies. These rarely reported long-term survivors did not die suddenly in early puppyhood, nor did they experience the progressive heart disease that eventually causes heart failure or fatal arrythmias by age two. Because adoption outcomes were readily available for the puppies in this litter, ECG, echocardiography, and cTnI were utilized in hopes of prognosticating for the seven surviving puppies. These seven remaining puppies have gone on to live in their adoptive homes for 5 years.

### 3.1. Echocardiography

Studies and cases where echocardiographic information for dogs with PMC are rare because most of the cases were reported in the late 1970s and the 1980s when the use of echocardiography in dogs was not commonplace. In two contemporary reports, echocardiography was used to evaluate cardiac function in dogs with clinical illness from parvovirus enteritis. In both reports, dogs who ultimately did not survive had significant reduction in left ventricular function based on echocardiographic parameters consistent with DCM phenotype (23, 24). Reports using echocardiography to characterize myocarditis of non-parvovirus etiologies and of dilated cardiomyopathy (DCM), the end stage result of myocarditis, describe parameters such as reduced left ventricular systolic function, left ventricular volume overload, dilation of the ventricles, and heteroechogenecity of the ventricular myocardium (25, 26). In this case report, puppy A4 and puppy A6 both had hyperechoic areas of the left ventricle that may have indicated ischemia on their initial echocardiograms at 11 weeks (Figure 3). Neither puppy had reduction of left ventricular function or dilation of the ventricles at that time. A4 had a repeat echocardiogram just under 2 years later and the hyperechoic areas in the left ventricle were no longer visible, no dilation of the ventricles was noted, and there was no reduction in left ventricular function indicating that the changes expected with advanced PMC were not present. The reason for the resolution of the hyperechoic, possibly ischemic, areas of the myocardium seen with echocardiogram on their initial examination is unclear. Perhaps there truly were regions of ischemia that resolved vs. the hyperechoic regions being a normal variation for this age of dog. Puppy A6 was not re-examined by echocardiogram, but 5 years later is still alive. All the other puppies had normal echocardiograms at 11 weeks of age indicating no evidence of myocarditis or the sequelae of myocarditis.

### 3.2. Electrocardiography

Parvoviral myocarditis has been characterized by ECG since cases were first recognized in the late 1970s. Because ECG was a readily available diagnostic, it was frequently utilized throughout the study periods described but examples commonly included single puppies and, in some cases, ECGs reported were recorded just days before puppies died. These studies report on several abnormalities seen on ECG which may be relevant to prognostication for PMC including ST segment elevation, QRS abnormalities and arrythmias (3, 11, 15, 18).

S-T segment elevation has been associated with myocardial ischemia in dogs (27). But while multiple studies have demonstrated that S-T segment elevation can be a sign of myocardial ischemia, S-T segment elevation as an indicator of cardiac damage specifically from PMC is an occasional but inconsistent finding (18). Conduction abnormalities, as seen with notched QRS complexes have been reported in a variety of cardiac diseases in human and canines, but again are occasionally but not consistently seen in puppies who later succumb to PMC (18, 28–30).

In addition to conduction abnormalities, sinus tachycardia, ventricular tachycardia and premature ventricular complexes have been occasionally reported in puppies that were diagnosed with PMC (3, 15, 18). The presence of ventricular tachycardia and premature ventricular complexes in puppies with PMC is consistent with reports of similar disease processes in dogs that result in death of cardiomyocytes and eventual fibrosis including myocarditis of other etiologies and idiopathic DCM (25, 31). In the few examples of puppies that eventually died due to PMC that are noted in the literature, arrhythmias were a precursor to eventual death due to cardiac failure (2, 3, 15).

The seven puppies evaluated in this study did not demonstrate any of the ECG abnormalities described above (Figure 3). For these puppies, ECGs were taken at nearly weeks old which is well-beyond the age where affected puppies have been described to have fibrosis, left ventricular dilation, and arrythmia and/or conduction abnormalities (2, 3, 15, 18). The lack of pathological ECG features alone does not rule out myocarditis or chronic heart changes. But taken as a part of the overall clinical and diagnostic picture, the absence of ECG pathology is a positive finding.

### 3.3. Cardiac troponin I

Cardiac troponin I is one of three subunits in a larger complex that mediates the starting and stopping of contraction within each sarcomere of a cardiomyocyte and can be released into the bloodstream when cardiomyocytes are injured (17). Cardiac troponin I has been used to detect both acute and chronic cardiomyocyte injury, and the level of cTnI correlates with the severity of cardiac damage detected by histology and with disease outcomes in a variety of canine cardiac diseases (17, 29, 32). In studies of dogs at high risk for heart disease, cTnI has proven to be a valuable tool for detecting myocardial damage and predicting outcomes for diseases such as myxomatous mitral valve disease, DCM and a variety of other acquired and congenital heart diseases (33–35).

The use of cTnI is also documented in dogs with CPV-2. Bastan et al. demonstrated that puppies with naturally occurring CPV-2 enteritis who died from the disease and had evidence of myocarditis at necropsy had significantly higher cTnI values (cTnI >0.8 ng/ml) compared with the values in dogs who either survived or died but had no cardiac involvement at necropsy (cTnI < 0.156 ng/ml) (36). Two other reports found a similar relationship between elevations in cTnI and non-survival in dogs with CPV-2 (23, 37).

In this case report, cTnI for all seven puppies fell within the range for healthy dogs at both the initial test and at the 1 month recheck, and among individuals cTnI values changed very little between the initial and final values (17) (Table 1). In addition, dogs A1 and A4 had cTnI levels that were either unchanged or lowered over a year and a half from the point when the first cTnI was taken, suggesting that progressive cardiac disease is not present (Table 1).

### 3.4. Limitations

The COVID-19 pandemic interfered with planned recheck echocardiograms and cTnI values for all puppies, except A1 and A4. A verbal check-in regarding the fate of the rest of the litter was substituted for recheck diagnostics for A2–3 and A5–7.

## 4. Conclusions

Within the litter of puppies described in this case report there were three different outcomes fitting with the three categories described: (1) one puppy died acutely at day 45 with evidence of parvovirus infection of cardiomyocytes (acute myocarditis), (2) another puppy died acutely at day 77 with histological evidence of extensive myocardial fibrosis and gross pathology evidence of DCM phenotype, and (3) the seven surviving puppies. The seven surviving puppies (A1–A7) have gone on to be sterilized and adopted with information about PMC. They are now nearly 5 years old. Two of the puppies were adopted even prior to the completion of all the initial diagnostic testing, indicating that even when prognosis is unclear, adopters who are provided with the available medical information regarding the puppies' health history were still willing to give these puppies a home. To the best of the authors' knowledge, this is the first time that the longer-term survival of puppies who came from a litter affected by PMC has been reported.

It is unclear what causes the variation of outcomes within a group of puppies infected with parvovirus in the perinatal period, but based on the findings of this case report it appears that not all puppies in litters affected by PMC will have a poor prognosis. Demonstrating that puppies do not have compromised cardiac health using echocardiography, ECG, and cTnI may be a useful platform for identifying those puppies with a more optimistic prognosis in PMC affected litters.

## Data availability statement

The original contributions presented in the study are included in the article/Supplementary material, further inquiries can be directed to the corresponding authors.

## Ethics statement

Written informed consent was obtained from the participant/patient(s) for the publication of this case report.

## Author contributions

BD: substantial contributions to the conception of the work, acquisition of samples, analysis, interpretation of data for the work, and drafted the manuscript. HK: contributions to the conception of the work, acquisition, analysis, and interpretation of data for the work. CA: interpreted the data, drafted the manuscript, and coordinated communication among the authors. AL and PB: provided pathology descriptions, analysis, and as well as pathology figures. SN: advised on the concept of the work, interpreted data, and drafted the manuscript. All authors critically reviewed the manuscript, provided revisions, and approved the submitted manuscript.
