# Supplementary material for: Case report: Long-term survival in puppies assessed with echocardiography, electrocardiography and cardiac troponin I after acute death in littermates due to parvoviral myocarditis
Source: Front Vet Sci. 2023 Aug 14;10:1229756. doi: 10.3389/fvets.2023.1229756 (PMC10462392; doi:10.3389/fvets.2023.1229756)
Supplement: Supplementary file 1 [file Table_1.pdf]

Supplementary Material

Case Report: Long-Term Survival in Puppies Assessed with Echocardiography, Electrocardiography and Cardiac Troponin I After Acute Death in Littermates Due to Parvoviral Myocarditis

Brenda Dines[1*], Heidi Kellihan[2], Carolyn Allen[1*], Alan Loynachan[3], Philip Bochsler[4], Sandra Newbury[1]

[1]University of Wisconsin-Madison School of Veterinary Medicine Shelter Medicine Program, Department of Medical Sciences, Madison, WI, USA

[2]University of Wisconsin-Madison College of Veterinary Medicine, Department of Medical Sciences, Madison, WI, USA

[3]University of Kentucky, Department of Veterinary Science, Veterinary Diagnostic Laboratory, Lexington, KY, USA

[4]University of Wisconsin-Madison School of Veterinary Medicine, Department of Pathobiological Sciences, Wisconsin Veterinary Diagnostic Laboratory, Madison, WI, USA

*** Correspondence:**
Corresponding Author(s):

Brenda Dines
reliefdinesdvm@gmail.com

Carolyn Allen
carolynallen@wisc.edu

1 Supplementary Figures and Tables

Table S1: Echocardiography results for A1-A7 on Day 70 and A1 & A4 on Day 640.

	**A1**	**A2**	**A3**	**A4**	**A5**	**A6**	**A7**	**A1**	**A4**
**Day 70**								**Day 640**	
**M-Mode Measurements**									
**IVSd (mm)**	5.13	6.97	5.74	6.56	6.26	6.82	6.58	12	11.79

**LVIDd (mm)**	24.31	25.23	23.18	21.33	24.21	24.53	24.29	35.08	36.24
**LVPWd (mm)**	5.54	5.95	5.23	5.85	4.41	6.46	5.74	7.08	10.26
**IVSs (mm)**	8.82	11.08	6.97	9.13	8.72	8/97	8.74	14.62	15.9
**LVIDs (mm)**	16	17.54	16	14.56	16.62	18.55	15.44	25.38	21.88
**LVPWs (mm)**	7.9	7.49	7.69	7.9	6.15	7.78	8.14	13.54	16.58
**EDV(Teich) (ml)**	21	23	18	15	21	21	21	51	55
**ESV(Teich) (ml)**	7	9	7	6	8	10	7	23	16
**EF(Teich) (%)**	66	60	61	63	62	51	69	55	71
**%FS (%)**	34	30	31	32	31	24	36	55	40
**SV(Teich) (ml)**	14	14	11	9	13	11	14	28	39
**LVIDdi**	1.344	1.359	1.292	1.158	1.396	1.254	1.253	1.333	1.324
**LVIDsi**	0.848	0.904	0.855	0.757	0.921	0.904	0.76	0.9	0.744
**2-D Measurements**									
**Ao Diam (mm)**	16.1	15.24	15.17	14.33	11.69	15.85	17.01	20.98	22.12
**LA Diam (mm)**	18.04	17.53	18.59	19.39	16.61	16.18	20.35	34.1	29.01
**LA/Ao**	1.12	1.15	1.23	1.35	1.42	1.02	1.2	1.63	1.31
**EF A-L LAX (%)**	55	51	61	56	62	66	71	70	63

Doppler Measurements									
**MV E Vel (m/s)**	0.86	0.77	0.83	0.64	0.71	0.57	0.56	0.86	0.71
**MV DecT (ms)**	87	120	98	90	51	88	104	157	90
**MV Dec Slope (m/s^2)**	9.9	6.5	8.5	7.1	13.8	6.5	5.4	5.5	7.9
**MV A Vel (m/s)**	0.4	0.49	0.60	0.35	0.52	0.39	0.4	0.58	0.51
**MV E/A Ratio**	2.16	1.58	1.39	1.82	1.35	1.47	1.42	1.49	1.39
**AV Vmax (m/s)**	1.22	0.75	1.04	0.8	1.08	0.93	1.05	1.41	1.25
**AV maxPG (m/s)**	5.91	2.27	4.34	2.57	4.70	3.45	4.38	7.98	6.2
**PV Vmax (m/s)**	0.73	0.77	0.88	0.66	0.76	0.88	0.84	0.95	0.75
**PV maxPG (mmHg)**	2.16	2.4	3.13	1.73	2.32	3.10	2.96	3.62	2.22
